# Sarcomere maturation: function acquisition, molecular mechanism, and interplay with other organelles

**DOI:** 10.1098/rstb.2021.0325

**Published:** 2022-11-21

**Authors:** Razan E. Ahmed, Takeshi Tokuyama, Tatsuya Anzai, Nawin Chanthra, Hideki Uosaki

**Affiliations:** ^1^ Division of Regenerative Medicine, Center for Molecular Medicine, Jichi Medical University, 3311-1 Yakushiji, Shimotsuke, Tochigi 329-0498, Japan; ^2^ Department of Pediatrics, Jichi Medical University, 3311-1 Yakushiji, Shimotsuke, Tochigi 329-0498, Japan

**Keywords:** cardiomyocytes, sarcomere, maturation

## Abstract

During postnatal cardiac development, cardiomyocytes mature and turn into adult ones. Hence, all cellular properties, including morphology, structure, physiology and metabolism, are changed. One of the most important aspects is the contractile apparatus, of which the minimum unit is known as a sarcomere. Sarcomere maturation is evident by enhanced sarcomere alignment, ultrastructural organization and myofibrillar isoform switching. Any maturation process failure may result in cardiomyopathy. Sarcomere function is intricately related to other organelles, and the growing evidence suggests reciprocal regulation of sarcomere and mitochondria on their maturation. Herein, we summarize the molecular mechanism that regulates sarcomere maturation and the interplay between sarcomere and other organelles in cardiomyocyte maturation.

This article is part of the theme issue ‘The cardiomyocyte: new revelations on the interplay between architecture and function in growth, health, and disease’.

## 1. Introduction

The heart is the first organ to function in a body and pumps blood throughout life. The heart adapts to produce sufficient force and match the demand as the body grows. Cardiomyocytes are the main force generators in the heart, and the sarcomere is the minimal unit to produce the force in cardiomyocytes. Cardiomyocytes change their morphology, structure, metabolism and physiology during the pre- and postnatal development; the whole process is called cardiomyocyte maturation [1,2]. Cardiomyocyte maturation is now considered the third and last phase of heart development and growth following specification and morphogenesis [3]. Sarcomeres undergo assembly to maturation in cardiomyocyte differentiation and maturation. Herein, we summarize the components that constitute sarcomeres, their assembly and maturation, and the regulatory mechanisms of the maturation process. Organelles in cardiomyocytes also structurally and functionally mature along with the sarcomere maturation, which would regulate sarcomere maturation or be regulated by sarcomeres. Here, we introduce the interactions between sarcomeres and other organelles in cardiomyocytes.

## 2. Constitution of sarcomere

**Figure 1.**
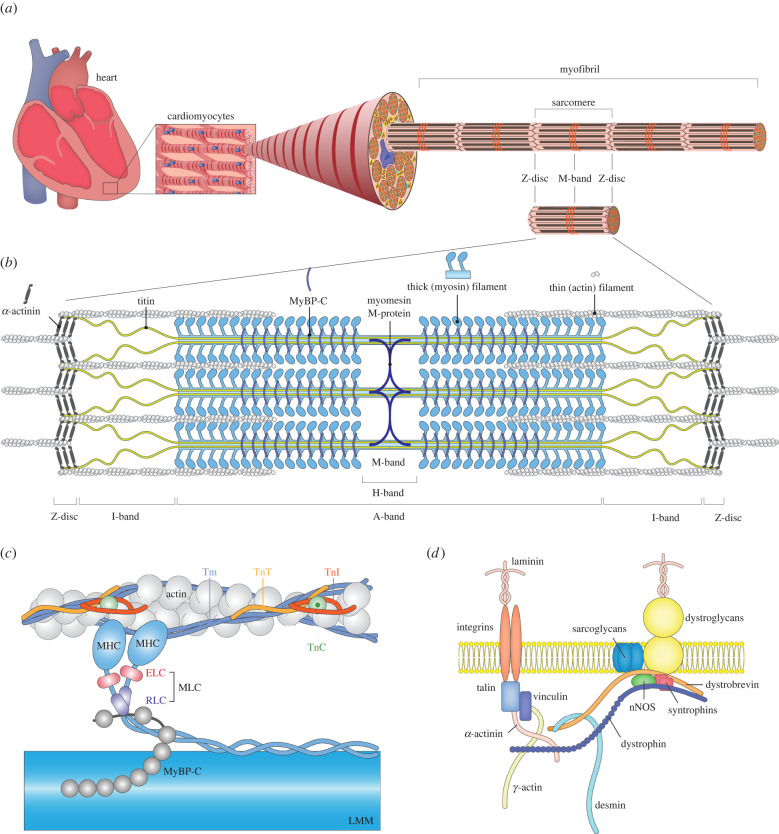
Structures supporting cardiac contractile force. (*a*) Hierarchical scheme of the cardiac structures. Cardiomyocytes generate the cardiac force. A bundle of myofibrils forms the muscle fibre in a single cardiomyocyte. Myofibrils are composed of the repeats of sarcomeres. Synchronous sarcomere contractions and relaxations cause heartbeats. (*b*) Structure of a sarcomere. Both ends of the sarcomere are Z-discs, and thin filaments, made of actin, are cross-linked to the adjacent sarcomeres through α-actinin. Thick filaments are composed mainly of myosin. Titin, one of the largest proteins in humans, spans from the centre of the sarcomere to the Z-disc, where it interacts with α-actinin. Microscopically, the dark A-band corresponds to the thick filaments, while the bright I-band consists only of the thin filaments and titin. (*c*) Detailed structures of the thin and thick filament. The main components of a thin filament are filamentous actin (F-actin), tropomyosin (Tm) and troponin complexes. Tm is a long dimeric coiled-coil protein that polymerizes from head to tail, covering most of the thin filaments except the Z-disc. A troponin complex consists of troponin T (TnT), troponin I (TnI) and troponin C (TnC), which interact with actin and Tm to regulate their calcium sensitivity while contracting. The thick filament has two segments, namely, myosin heads and light meromyosin (LMM). Two myosin proteins dimerize through their long tails and further compose an LMM subfragment with other myosin tails. The myosin head is composed of two globular multi-domain heads that bind to actin and hydrolyse ATP and necks that are stabilized by myosin essential light chains (ELCs) and regulatory light chains (RLCs). The C-terminal domain of myosin-binding protein C (MyBP-C) is anchored to titin and myosin LMM, while the N-terminal domain interacts with myosin and possibly actin. (*d*) Scheme of costamere. Costameres anchor sarcomeres to sarcolemma and transduce signals and forces between inside and outside of the cardiomyocytes through the extracellular matrix.

Sarcomeres longitudinally repeat to form a myofibril that serves as the contractile apparatus of cardiomyocytes (figure 1*a*). Thus, a sarcomere is the minimal contractile unit, mainly consisting of thin and thick filaments. The thick filaments are composed of numerous myosin heads that attach to actin in the thin filaments that create actin-myosin cross-bridges (figure 1*b*). Lines and bands of different electron densities form Z-discs and M-, A- and I-bands because of the structural components. Both ends are demarcated by Z-discs, which are thin discs with a high electron density. An electron-dense A-band is formed owing to the presence of parallelly aligned thick filaments, primely composed of myosin, at the middle of a sarcomere [4]. Less electron-dense bands form I-bands, composed of actin and titin between A-band and Z-discs. The H-zone is a low electron density region in the middle of the A-band in which the thick filaments are the only longitudinal elements. At the very central region of the H-zone, an electron-dense line, M-band, serves to arrange thick filaments into A-bands.

### (a) Thick filament

The major component of a thick filament is myosin II, which contains four light chains and two heavy chains, myosin light chain (MLC) and myosin heavy chain (MHC), respectively (figure 1*c*). Different MLC and MHC proteins form skeletal, cardiac and smooth muscle myosin. α-MHC (encoded by *Myh6*) and β-MHC (encoded by *MYH7*) are the predominant MHC expressed in mice and humans, respectively in the adult heart. Two heavy chains form a complex of two heads and a rod. The rod is an α-helical coiled-coil structure, which is crucial for the complex formation, and connects to two globular heads via flexible hinges [5,6]. The high-resolution crystal structure of the myosin head revealed the active site for adenosine triphosphate (ATP) hydrolysis, the binding of two MLCs to an extended α-helix just before the tail domain and the binding site for actin filaments [7]. MLC has two distinct subtypes, essential and regulatory light chains (ELC and RLC, respectively). In ventricular cardiomyocytes, MLC2v and MLC1v are RLC and ELC, respectively [7]. *MYL2* and *MYL3* genes encode these MLCs, respectively. The cardiac isoform of myosin-binding protein C (MyBP-C), encoded by *MYBPC3*, binds to titin (encoded by *TTN*) and light meromyosin (LMM, the tail region of myosin) in the A-band via its C-terminal domain [8,9]. In the M-band, the myomesin family, namely, myomesin, M-protein/myomesin-2 and myomesin-3, encoded by *MYOM1*, *MYOM2* and *MYOM3*, respectively in humans [10–12], acts as a structural sarcomere stabilizer by cross-connecting thick filaments [13–15].

### (b) Thin filament

The backbone of a thin filament is filamentous actin (F-actin), which is predominantly encoded by α-cardiac actin (*ACTC1*) in the heart (approx. 80%) [16]. The thin filament of striated muscles has two additional functional components, namely, the tropomyosin (Tm) and troponin (Tn) complex, which modulate sarcomere function in addition to the actin filament backbone (figure 1*c*). Two Tm molecules form a long thin, stranded α-helical coiled-coil filament that occupies two grooves of the actin filament. Three subunits of Tn—troponin T (TnT), troponin I (TnI) and troponin C (TnC)—form a Tn complex. The TnT molecule is a binding subunit to Tm, whereas the other two subunits are globular and link TnT to actin. Moreover, TnI and TnC are the regulator domain of Tn in response to Ca^2+^. TnI consists of two parts, one is a part to form a complex with TnT and TnC and the other inhibits sarcomere contraction by interfering with the Tm on the actin filament [17,18]. The C-terminal of TnI protein constrains the Tm position on the actin filament, and TnI and Tm sterically block myosin from interacting with actin in the absence of Ca^2+^. Contrastingly, Ca^2+^ binding leads to a conformational change of TnC, which subsequently releases TnI and Tm from the myosin-binding region of the actin filament, allowing myosin to bind to actin, thereby resulting in sarcomere contraction [17–20]. A myosin produces the power stroke to slide the actin filament while cross-bridging between actin and thick filaments with ATP hydrolysis [21,22]. The power stroke slides the actin filament past the myosin; hence, reducing the distance between the Z-discs while thick and thin filaments remain at the same length.

### (c) Z-disc

At the Z-disc, α-actinin proteins form antiparallel homodimers and cross-link actin filaments to adjacent sarcomeres, thereby framing a lattice-like structure that stabilizes the muscle contractile apparatus [23,24]. Encoded by *ACTN2*, α-actinin-2 is a major isoform in the cardiac muscle. Encoded by *TCAP*, telethonin is also located in the Z-discs of a sarcomere and plays an important role in the sarcomere assembly as it joins titin [25,26]. Titin provides passive elasticity to muscles and is also essential for maintaining structural sarcomere integrity. A single titin molecule spans the half sarcomere, binding to the Z-disc and the thin filament at its N-terminus and the thick filament and M-band with its C-terminus. Among three cardiac titin isoforms, N2B titin is the most abundant in the left ventricles [27].

A costamere anchors the Z-disc of myofibrils to the sarcolemma (figure 1*d*). The costamere is a sub-membranous structure in striated muscles and is composed of two major protein complexes, the dystrophin-glycoprotein complex and the vinculin-talin-integrin complex [28]. Desmin is a major intermediate filament protein that links between costameres and Z-discs [29]. It also connects Z-discs to Z-discs, mitochondria and the nucleus [30]. Generally, costameres are thought to transmit forces bidirectionally between the sarcomeres and the sarcolemma [31–33]. Additionally, they function as important centres of intracellular signalling, in which integrin-filamin and ankyrin-desmin are also involved [34,35]. The signalling is also bidirectional between the extracellular environment and the intracellular signalling network (outside-in/inside-out signalling) [36,37]. Many proteins associated with Z-discs and costameres have been identified, and mutations in many of those have been associated with cardiomyopathy and skeletal myopathy in humans and mice [33,38–40].

## 3. Sarcomere assembly

**Figure 2.**
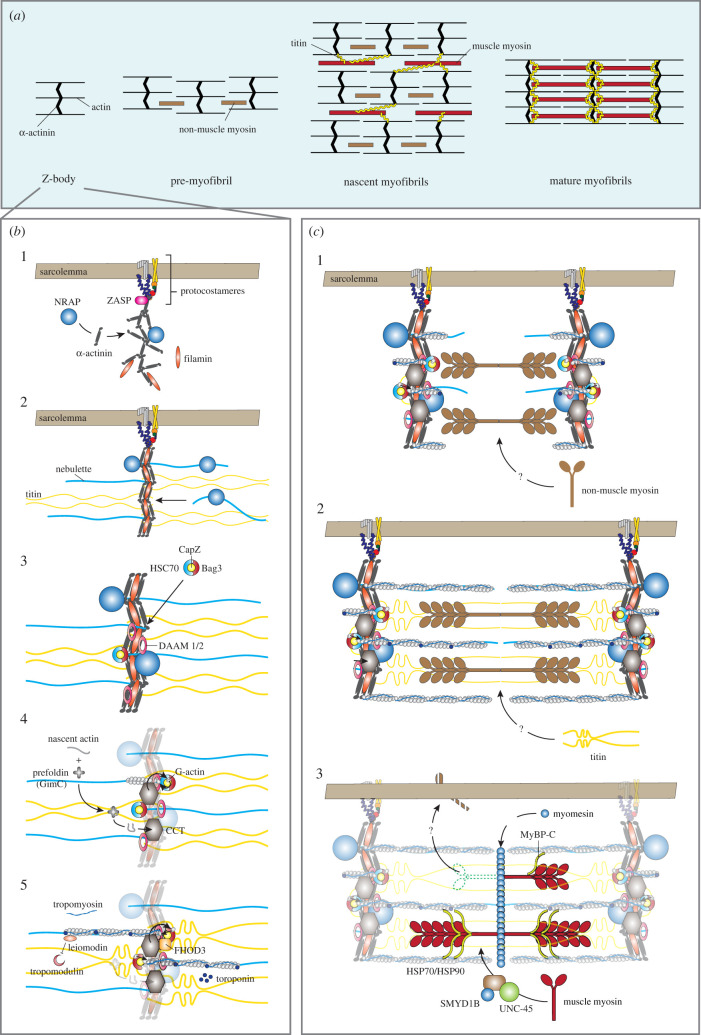
Sarcomere assembly. (*a*) Schematic diagram of the pre-myofibril model. Z-bodies form pre-myofibrils with non-muscle myosin. Pre-myofibrils further connect to become nascent myofibrils. A wide array of myofibrils aligns with the integration of titin and the replacement of non-muscle myosin with muscle myosin. (*b*) Z-body and thin filament assembly: 1. at the proto-costamere (precursor of costamere), the Z-body forms following the Z-disc alternatively spliced PDZ-motif protein (ZASP) recruitment to the sarcolemma. ZASP recruits proteins, e.g. α-actinin, nebulin-related anchoring protein (NRAP) and filamin, to assemble the Z-body; 2. nebulin/nebulette and titin are incorporated into the Z-body; 3. BAG3 simultaneously localizes CapZ to the Z-body with dishevelled associated activator of morphogenesis 1 and 2 (DAAM1/2); 4. prefoldin (GimC) delivers the nascent actin to chaperonin-containing T-complex protein 1 (CCT), which folds it into its final conformation. CCT then transfers the folded G-actin to BAG3. Finally, formin proteins (DAAM1/2 and FHOD3) polymerize G-actin to F-actin; and 5. troponin complex and tropomyosin are incorporated into the I-band by an unknown mechanism. Leiomodin stabilizes the growing fibres and competes with tropomodulin not to stop actin polymerization. Leiomodin detaches when actin filaments reach mature length, and the filaments are capped by tropomodulin. (*c*) Pre-myofibril model of thick filament assembly: 1. non-muscle myosin II is incorporated between the Z-bodies to form pre-myofibrils; 2. titin is integrated into pre-myofibrils to align nascent myofibrils; and 3. Muscle myosin replaces non-muscle myosin, and M-line proteins and MyBP-C are incorporated to complete the assembly of myofibrils.

Sarcomeres are the centrepieces of force generation in the cardiomyocytes, and approximately 200 sarcomere-associated proteins are annotated in the gene ontology database (GO:0030017, Sarcomere). Sarcomere formation starts when cardiomyocytes differentiate and continue through early postnatal life, and then the sarcomere structure is maintained throughout life. Over the last three decades, several initiation and propagation models of the sarcomere assembly have been proposed, which include models of pre-myofibril, template sarcomere assembly and independent subunit sarcomere assembly [41]. Among these concepts, one in common is that the Z-body, the precursor of Z-disc, is assembled first, then thin filaments form as a part of a Z-body or on Z-disc after forming the myofibrils (figure 2*a,b*) [42]. Finally, thick filaments replace non-muscle myosin, assembled *in situ*, or separately assembled and incorporated into sarcomeres [41].

### (a) Z-disc assembly

One of the key structures of the initiation for the assembly is the Z-body, also known as the I-Z-I brush (figure 2*a*). This structure consists of α-actinin and actin. Pre-myofibrils, stress fibre-like structures of Z-bodies and non-muscle myosin II, form in the cell periphery via either an integrin-dependent mechanism or as latent complexes forming throughout the myocyte [43–45]. Z-bodies are recruited into the proto-costameres, areas of high integrin concentration of the sarcolemma, by Z-disc alternatively spliced PDZ-motif protein (ZASP) [42,46–48]. Then, ZASP recruits α-actinin, nebulin-related anchoring protein (NRAP) and filamin, which in turn anchors titin to the Z-disc through nebulette [47,49,50]. Z-bodies fuse to form the wide lateral arrays of mature Z-discs.

### (b) Thin filament assembly

The thin filament assembly starts on a Z-body or Z-disc (figure 2*b*) [42]. The monomeric form of actin, the globular actin (G-actin), polymerizes to form the filament (F-actin) from the Z-disc edge, with their barbed ends toward the Z-disc end. The actin polymerization step is regulated by a series of proteins. CapZ (the muscle isoform of capping protein; also known as β-actinin), α-actinin and other proteins block the barbed ends of the actin filaments in the Z-disc and contribute to initiating their polymerization [23,51]. CapZ is recruited to the Z-disc with BAG3, one of the Bcl2-associated athanogene (BAG) family proteins, before the actin polymerization. Prefoldin (GimC) enhances actin folding and prevents G-actin aggregation [52]. Chaperonin-containing T-complex protein 1 (CCT), a Z-disc protein, folds the actin to the final conformation and transfers the folded actin to BAG3 [53,54]. The rho GTPase-binding formin homology protein family, such as dishevelled associated activator of morphogenesis 1 and 2 (DAAM1/2) and formin homology 2 domain-containing 3 (FHOD3), regulates actin polymerization to F-actin. Tropomodulin caps and limits the thin filament, while leiomodin competes with tropomodulin until the thin filament reaches its mature length [55]. BAG3 family proteins represent an evolutionarily conserved group of heat shock 70 kDa protein (HSP70)/heat shock cognate 71 kDa protein (HSC70) binding co-chaperones [35,56]. BAG3 binds to the actin capping protein CapZ, which ensures the stability of the actin network and its proper Z-disc anchorage, together with HSC70 [57]. BAG3 also involves selective sarcomere protein autophagy to keep sarcomere integrity [58].

### (c) Thick filament and myofibril assembly

Z-body and thick filament assembly to a myofibril differ depending on the assembly models. Across the models, titin is considered the main scaffold. Additionally, full-length titin, β-cardiac myosin and α-actinin are required for sarcomere assembly in human cardiomyocytes [59], although titin is not essential for Z-body or the thick filament formation [60,61]. In the pre-myofibril model (figure 2*c*), a widely supported one, the Z-bodies first formed as the pre-myofibrils with non-muscle myosin II. They lack titin, and the distance between them ranges from 0.3 to 1.4 µm [44]. Then, titin addition and thin filament elongation process pre-myofibrils to nascent myofibrils, and the distance increases to 1.8–2.5 µm [44]. Finally, muscle myosin replaces non-muscle myosin and M-line proteins and MyBP-C are incorporated to complete the myofibril formation [43,44,62–64]. Protein chaperones, uncoordinated mutant number 45 (UNC-45), SET and MYND domain-containing proteins (SMYD1B), HSP70, and HSP90 regulate myosin folding and thick filament assembly [65]. The template sarcomere assembly model is similar to the pre-myofibril model. Pre-myofibrils form first, and muscle myosin and M-band proteins are folded and incorporated as thick filaments *in situ* on titin that is extended from Z-bodies by the chaperones. In the independent subunit sarcomere assembly model, pre-assembled thick filaments are linked to the Z-bodies via titin [66,67].

## 4. Maturation process of sarcomere in cardiomyocytes

Sarcomere maturation is a constant and gradual process to develop a complete ultrastructure in cardiomyocytes so that cardiomyocytes can generate sufficient contractile forces for blood circulation. The sarcomeres are still disorganized as cardiomyocytes differentiate in an early embryo. The sarcomeres organize and align well to form parallel myofibrils throughout the cardiomyocytes when more maturation processes proceed [2]. Part of the organization and alignment can be explained by sarcomere assembly as previously described. During the sarcomere maturation (from late embryonic to the adolescent stages), new sarcomeres are continuously added in alignment with pre-existing myofibrils, both longitudinally and laterally, to expand myofibrils [3]. *In vitro* studies revealed that sarcomeres are added to the lateral margins of myofibrils to increase the number of myofibrils, whereas the addition of sarcomeres to the edges of existing myofibrils increases their length [68,69]. Under longitudinal stretches, sarcomeres can also be added to the middle of myofibrils [69]. The distance between Z-discs increases from approximately 1.7 µm to approximately 2.2 µm when matured [70,71]. Mechanical force regulates the sarcomere maturation through vinculin [72]. Once sarcomeres mature, continuous maintenance occurs by replacing sarcomere proteins with newly synthesized ones. This maintenance process occurs throughout life [64]. Moreover, the composition of sarcomeres changes owing to the isoform switching of sarcomere proteins, leading to physiological property changes in the sarcomeres. Additionally, M-bands are difficult to observe in fetal cardiomyocytes. With the isoform switch and increased M-band protein expression, M-bands become distinct [73]. Here, we summarize two aspects of the sarcomere maturation process, isoform switching and its effects on sarcomere physiology.

### (a) Isoform switching

The isoform switching of sarcomeric proteins in the troponin complex, MHC, MLC and titin from fetal to adult ones through transcriptional changes or alternative splicing is the essential element of myofibril maturation. For instance, the slow skeletal muscle isoform of troponin I (ssTnI, encoded by *TNNI1*) is predominantly expressed in fetal hearts, and the isoform turns to cardiac ones (cTnI, encoded by *TNNI3*) after birth [74,75]. Two different MHC isoforms are alternately expressed during heart development. β-MHC (encoded by *Myh7*) is the major isoform in fetal cardiomyocytes in mice, while α-MHC (encoded by *Myh6*) is the adult isoform [76]. Conversely, α-MHC is expressed in fetal hearts, and β-MHC is expressed in adult ones in humans [1,77]. Transcriptional regulation also switches the MLC isoforms in ventricular cardiomyocytes. Primitive fetal ventricular cardiomyocytes express both MLC2v (the ventricular isoform of MLC, encoded by *MYL2*) and MLC2a (the atrial isoform of MLC, encoded by *MYL7*), whereas MLC2v becomes the predominant isoform once ventricular cardiomyocytes mature. Contrastingly, atrial cardiomyocytes have no MLC isoform switching [78,79]. The coding gene of titin (*TTN*) has 363 exons in humans, and the alternative splicing of *TTN* creates stiff N2B and more compliant N2BA isoforms [80,81]. Among N2BA isoforms, at least four different isoforms were reported in the heart (3220–3710 kDa in rats) [81]. Fetal N2BA isoforms (3590 kDa and 3710 kDa), which are larger than adult N2BA isoforms (3220 kDa and 3390 kDa), exist in the fetal heart. These larger fetal isoforms have a longer middle immunoglobulin domain, which works as a molecular spring; thus, they are the most compliant titin isoforms. The fetal cardiac titin isoforms quickly disappear after birth, and adult N2BA and N2B isoforms are upregulated [80,81]. N2B (2970 kDa) is the dominant titin isoform in the adult heart. ACTC1 is the predominant actin isoform in the adult heart; however, ACTA1 (skeletal) and ACTA2 (smooth muscle aorta) are also expressed. These two isoforms are expressed higher in embryonic hearts than in adult hearts and upregulated in disease conditions [82–84]. Like other sarcomere components, M-bands also undergo the isoform switch from fetal myomesin (embryonic heart [EH]-myomesin, a splicing variant of *MYOM1*) to mature one lacking the EH domain, including the M-protein/*MYOM2* upregulation [13,85]. This isoform switch is associated with the emergence of the clear M-bands under electron microscopy [86]. These sarcomere isoform switches are considered to occur upon birth [3]. Quick downregulation of *Myh7* and *Tnni1*, and upregulation of *Myh6* and *Tnni3* were observed after birth with transcriptome analysis in mice [87].

During the cardiomyocyte maturation process, dynamic transcriptional changes were observed, and thousands of genes, not only sarcomere genes but structural, ion channel, and mitochondrial/metabolic genes, are upregulated [87–90]. These genes include the aforementioned genes, *TCAP*, *MYOM2* and *TNNI3*, as the sarcomeric genes [87].

Sarcomere isoform switches are coupled with the maturation process; however, they also occur in heart failure. Hence, fetal isoforms revert to appear. The ratio of α-MHC and β-MHC is often used as a heart failure marker in the disease models, although the impact of the isoform switch in human failing hearts remained debatable [16,91]. The N2BA isoform of titin and EH-myomesin also re-express in failing hearts [92,93].

### (b) Sarcomere maturation and changes in physiological properties

Cardiomyocyte maturation is a dynamic process in which sarcomeres and other cellular elements that interact with sarcomeres reach the adult level of maturity. For example, sarcomeres regulate cellular morphology. Adult cardiomyocytes exhibit a rod shape with an approximately 7 : 1 length-to-width ratio [94]. Cardiomyocytes lacking *Myh6* or *Actn2* retained their elongated morphology but the cell width was drastically decreased with reduced transverse tubules (t-tubules) and disrupted mitochondrial morphology using a genetic mosaic model in mice [95,96].

Isoform switches of sarcomere proteins change the way of interactions between sarcomeres and other cellular components and lead to physiological adaptation to adult contractility demands, along with other cardiomyocyte property maturation. The transition from high energy demanding α-MHC to the more forceful and energy sparing β-MHC in mature ventricular cardiomyocytes ensures optimum contractility [91,97]. Their differences in ATPase activity determine the speed of sarcomere contraction [98]. A small percentage of α-MHC significantly enhances cell contractility in rat cardiomyocytes [99]. Moreover, the expression ratio imbalance of α- and β-MHC is linked to cardiomyopathy, atrial fibrillation and heart failure. Therefore, tight regulation of the two isoforms ratio is required. Similar to the isoform switch of MHC, isoform switches of MLC, titin and myomesin lead to changes in physiological properties and/or calcium sensitivity. Ventricular cardiomyocytes of the early mouse embryo express MLC2a, while MLC2v takes over by embryonic day 14.5 [100], which might be associated with a ventricular action potential morphology [101]. Moreover, MLC2v is phosphorylated by MLC kinases, which increases the step size of myosin and increases the contraction force [102,103]. The isoform switch of titin changes sarcomere compliance [104]. Titin provides passive sarcomere resistance. N2B is stiffer, while N2BA is more compliant between the two adult titin isoforms [105]. The N2B isoform is predominant and N2BA is less expressed in healthy hearts [105].

The distance between Z-discs (sarcomere length) becomes approximately 2.2 µm during the sarcomere maturation process, in which sarcomere has the best performance. Sarcomere length is tightly coupled with force generation, which is the molecular basis of the Frank-Starling Law [106–108]. Sarcomere and thin and thick filament lengths determine the overlaps between them and the number of myosin heads available to bind to actin and generate tension. Sarcomere length is determined by the balance between its active force and the pre- and post-loads [37], and blood pressure is considered one of the causes for the sarcomere length increase after birth [109]. Blood pressure increases owing to an increased systemic vascular resistance after birth [110]. With time, repeated loads increase sarcomere length to adapt to the growing demand [111].

## 5. Molecular mechanisms regulating sarcomere maturation

The molecular mechanisms that control cardiac myofibrillar maturation remained inadequately understood although sarcomere assembly and maturation are studied as previously summarized. Isoform switches and sarcomere protein expressions are regulated by transcription and translation. Here, we summarize the reports that highlighted the roles of possible regulatory molecules for sarcomere formation through transcription and translation, including indirect evidence of cardiomyopathy phenotypes with their alteration, indicative of sarcomere formation disruption.

### (a) Transcription factors

Serum response factor (SRF) is a critical transcription factor that regulates several aspects of growth and muscle differentiation. Cardiac-specific knockout of SRF in mice embryos severely disrupted cardiac sarcomeres causing lethal contractility defects around embryonic day (E) 10.5–13.5 [112,113]. Adeno-associated virus (AAV)-based genetic mosaic analysis showed that SRF regulates the cardiomyocyte maturation in neonatal mice hearts in a tightly controlled, time-specific manner [95]. Either increased or decreased SRF levels in neonatal mice hearts disrupted normal sarcomere maturation, which in turn impaired several cardiomyocyte maturation aspects, suggesting sarcomeres as a key cardiomyocyte maturation regulator [95].

The major coactivators of SRF are the myocardin family, namely, myocardin and myocardin-related transcription factors (MRTFs) A and B [114]. Cardiac-specific double knockout of MRTFA/B in mice caused sarcomere disarray and decreased cardiac functions. Most of the mutant mice died in the first month of life and surviving mice displayed severe ventricular dilation and reduced cardiac function, suggesting the importance of MRTFA/B in sarcomere assembly [115]. Indeed, the α-actinin–α-actin–MRTF–SRF signalling axis was probed to involve sarcomere and cardiomyocyte maturation using the AAV-based mosaic assay [96]. The monomeric form of β-actin (G-actin) retains MRTFs in the cytoplasm to inhibit SRF activation, while F-actin does not [116]. The cardiac-specific α-actin isoform, ACTC1, possesses the same ability, and α-actinin orchestrates the assembly of α-actin to F-actin, reducing G-actin levels. In turn, MRTFs enter the nucleus and activate SRF-dependent gene expression programmes to further enhance sarcomere assembly in a positive feedback manner. Disruption of α-actinin increased monomeric α-actin isoform (G-actin), thereby retaining MRTFs in the cytoplasm [96]. Another SRF-binding transcription coactivator is homeodomain-only protein, which is involved in cardiomyocyte hypertrophic response and myofibrillar switching to more mature isoforms [117].

GATA-binding protein (GATA) 4 and 6 are important transcription factors for normal heart development. They share partly redundant functions. Cardiac-specific GATA4 ablation resulted in progressive cardiac dilatation and functional deterioration. GATA6 knockout caused a significant reduction in heart size with no significant effect on cardiac function, and both GATA4 or GATA6 knockout mice had decreased cardiomyocyte hypertrophy in response to pressure overload [118,119]. Fetal double knockout of GATA4 and 6 caused severe ventricular dilation and decreased cardiac function, and death by 16 weeks of age [119].

### (b) Nuclear receptors

Nuclear receptors play an important role in cardiac development, homeostasis and disease pathogenesis in the heart. Among the nuclear receptor superfamily, thyroid hormone receptors (THRs), oestrogen-related receptors (ERRs), glucocorticoid receptors and peroxisome proliferator-activated receptors (PPARs) are known to be involved in cardiomyocyte maturation.

The thyroid hormone has a critical role in cardiac development and cardiovascular physiology [120]. Tri-iodothyronine (T3) is important for titin and MHC isoform switching, along with sarco/endoplasmic reticulum Ca^2+^-ATPase (SERCA) 2a expression [121–123]. Two THR isoforms, THRa and b, are found in the heart. Of the two, THRa mediates primary T3 effects on sarcomere maturation. THRa knockout mice showed decreased contractile functions of the papillary muscle and lower levels of *Serca2a* and *Myh6* transcripts similar to that observed in hypothyroid mice [124]. THRa mutation in zebrafish also caused decreased contractility, with abnormal sarcomere organization [125]. Cardiac-specific expression of D337T mutant or a dominant-negative form of THRb in mice caused bradycardia and decreased cardiac function and left ventricular hypertrophy with ageing, suggesting the later roles compared to THRa [126,127].

ERRs are known to participate in cardiac maturation and postnatal regulation of mitochondrial development and function [128]. Postnatal cardiac-specific ERR*α*/*γ* knockdown in mice caused cardiomyopathy, decreased mitochondrial function, reduced expression of structural, ion channel/transporters, and mitochondrial and calcium handling genes. Prenatal ERR*α*/*γ* knockdown mice exhibited left ventricle thinning along with disrupted mitochondrial structure and function, and all mice died within 24 h of birth [128].

Glucocorticoid receptors are important for normal fetal heart maturation. Mutation of the *Nr3c1* gene encoding glucocorticoid receptors in mice disrupted sarcomere organization, decreased cardiomyocyte alignment and impaired calcium handling [129].

PPARs have three different isoforms, namely, *α*, *β*/*δ* and *γ*. Once activated, they bind to the retinoid X receptor to conduct their transcriptional activity. The PPAR family generally functions in regulating glucose and lipid metabolism. However, each member seems to have distinct functions [130]. Abnormal PPAR levels in the heart are linked to many diseases. Decreased PPAR*α* and increased PPAR*γ* in the right ventricle are linked to arrhythmogenic right ventricular dysplasia [131,132]. Decreased PPAR*α* is also associated with developing pressure-induced cardiac hypertrophy [133]. PPAR*γ* is critical for normal heart development. PPAR*γ* gene deletion in mouse embryos caused severe myocardial dysplasia at E10, leading to embryonic lethality [134]. However, two later studies reported different pathological phenotypes of PPAR*γ* gene knockout, hypertrophic and dilated cardiomyopathy (DCM) [135,136].

PPAR*γ* coactivators *α* and *β* (PGC1*α*/*β*) serve as a coactivator of nuclear receptors, not only for PPARs but for THRs and ERRs [137] and are known for their role in metabolism and mitochondrial biogenesis [138]. Recently, PGC1/PPAR signalling was reported as essential for normal contractile function, cell hypertrophy and calcium handling maturation, partly through a transcriptional factor, Yes-associated protein 1 (YAP1) [139].

### (c) MicroRNAs

MicroRNAs (miRNAs) are a large group of small, non-coding RNAs, which regulate different cardiomyocyte aspects. miRNAs and transcription factors regulate each other in developing hearts, and they control cardiac gene expression together [140]. An miRNA can affect the translation of hundreds of messenger RNAs [141]. Dicer1 is an essential endonuclease for miRNA processing and maturation. Thus, postnatal cardiac-specific Dicer1 deletion leads to a global miRNA loss in cardiomyocytes with marked left ventricular dilation and impaired cardiac function. At the cellular level, cardiomyocytes showed decreased contractile proteins and myofibrillar disarray [142]. Dicer1 inactivation using a pan-cardiomyocyte Cre driver at mid-gestation in mice leads to embryonic lethality between E14.5 and E16.5. The mutant mice had severe myocardial wall defects with decreased cell proliferation and increased apoptosis and contractile protein misexpressions. These results imply the importance of miRNAs in sarcomere development and maturation.

Certain miRNAs were shown to be more important in cardiac development and maturation in addition to the global miRNA functions in the heart. MicroRNA-1 (miR-1) is highly expressed in developing hearts. Two copies of miR-1 are found in most mammals, namely, miR-1-1 and miR-1-2. miR-1 directly acts on the ERR*β* to repress fetal cardiac gene programmes and control the transition from the prenatal to the neonatal stage. miR-1 knockout mice resembled the control mice at P2.5. Afterwards, they suffered DCM and failed to survive past P17 [140]. However, another study reported that miR-1 knockout was fatal by P10 [143]. Deletion of miR-1–1 alone also decreased sarcomere organization, with mild ventricular dilatation and conduction abnormalities [143]. MicroRNA133a1 and 2 (miR133a1/2) are muscle-specific miRNAs. Double knockout mice exhibited right ventricular thinning by E12.5 but were otherwise normal. Then approximately, half of them developed severe ventricular dilatation and ventricular septal defects and became lethal by P1. Surviving mice grew but had DCM and fibrosis. Transmission electron microscopy showed significant sarcomere fragmentation and disorganization along with disrupted Z-discs [144].

### (d) RNA-binding proteins for alternative splicing

RNA-binding motif protein (RBM) 24 is an RNA-binding protein that is exclusively expressed in the heart and skeletal muscles and regulates the alternative splicing of genes that are important for cardiac development, such as certain transcriptional factors, cytoskeleton proteins, and ATPase gene family members [145]. Abolishing RBM24 function in zebrafish embryos resulted in decreased sarcomere proteins and reduced heart contractility [146]. Serine/threonine kinase (STK) 38 regulates RBM24 stability through phosphorylation during sarcomere development. STK38 deficiency reduces sarcomere proteins and causes sarcomere disarray [147]. Other RNA splicing factors, RBM20 and heterogeneous nuclear ribonucleoprotein U (hnRNPU), are also important for sarcomere maturation. RBM20 regulates titin splicing, and its deletion caused pathological titin isoform expression and DCM phenotype in rats [148]. More importantly, *RBM20* mutations are found in familial DCM [149]. Cardiac-specific hnRNPU knockout mice exhibited impaired sarcomere dynamics, abnormal calcium handling, severe DCM and lethal heart failure within two weeks of birth [150]. These findings highlight the importance of alternative splicing for sarcomere regulation and cardiomyopathy development.

## 6. Relationships to other organelles

**Figure 3.**
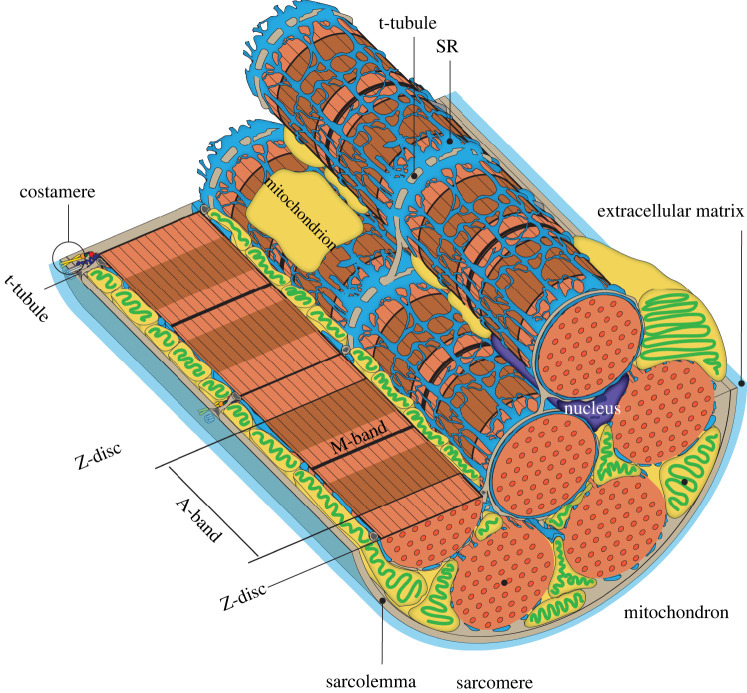
Interactions between sarcomeres and other organelles: sarcolemma, the plasma membrane of a cardiomyocyte, covers the myofibres and other organelles. Myofibres are composed of many myofibrils, and the cardiomyocytes appear to be striated because sarcomeres in myofibrils have light and dark bands. The costamere, a major muscle multi-protein complex on the sarcolemma, anchors and coordinates myofibrils with the sarcolemma and extracellular matrix. T-tubules, the invagination of the sarcolemma, are located transversely to the sarcomere and form close contact with the sarcoplasmic reticulum (SR), while SR forms a mesh-like structure to cover the myofibres. The close association of t-tubules with SR promotes a rapid calcium increase through calcium-induced calcium release upon action potential formation. Mitochondria reside next to the myofibres along t-tubules and SR, and several proteins have been shown to exist between the mitochondria and SR, which are known as mitochondria-SR contact sites. These structures maintain proximity between organelles and allow mitochondria to exert significant Ca^2+^ fluctuations at each action potential. Furthermore, these SR-mitochondria-t-tubule interactions modulate the calcium and ATP that regulate sarcomere contraction.

A more comprehensive approach, which considers other cellular organelles, is needed to deepen our understanding of sarcomere maturation. Not only sarcomeres but also organelles are highly organized and linked with each other to facilitate efficient cardiomyocyte beating (figure 3). Molecular and physical interchanges between sarcomeres and other organelles remain largely unknown. Hence, elucidating these interactions and their effects on the maturation of sarcomeres and other organelles may provide a further understanding of cardiomyocyte biology and cardiac diseases and be the missing step in our attempts to produce mature, adult-like cardiomyocytes *in vitro*. Here, we summarize how organelles interact with sarcomeres (figure 3).

### (a) Mitochondria

The mitochondria are the powerhouse of the eukaryotic cell. They produce the energy needed to sustain heart beating among other functions in the heart. Mitochondrial activity in cardiomyocytes is particularly strong compared to that in skeletal and smooth muscles [151]. A recent three-dimensional electron microscopy study revealed different mitochondrial networks, morphology and interactions with other cellular components compared to the other muscles, implying that the energy demand regulates mitochondria [152]. The decreased mitochondrial function causes cardiac dysfunction and leads to cardiomyopathy with sarcomere disarray, suggestive of mitochondrial involvement in sarcomere organization or homeostasis [153–155].

The mitochondria need to meet the demand from sarcomeres even during myofibrillogenesis [156,157]. Embryonic cardiomyocytes mainly use glycolysis, while adult ones rely on fatty acid beta oxidization, which is coupled with the mitochondrial morphological transition from reticular to lattice [158]. Lattice mitochondria distribution allows close contact and regular alignment of mitochondria with sarcomeres, an important feature for an efficient ATP delivery to sarcomere from mitochondria [159].

Cytoskeletons have important roles to keep mitochondria arrangement with sarcomeres. The proteolytic treatment of trypsin disrupted the cytoskeleton and disorganized the alignment of mitochondria in permeabilized cardiomyocytes, suggesting that the cytoskeleton maintains the mitochondrial arrangement in cardiomyocytes [160,161]. Later, non-sarcomeric cytoskeletons, e.g. desmin intermediate filaments, were revealed to anchor mitochondria to sarcomere [162,163]. Desmin mutations are associated with cardiomyopathy, supporting its crucial roles in maintaining proper mitochondrial distribution and sarcomere integrity [30,164]. The coupling of mitochondria to the cytoskeleton is also necessary to facilitate nucleotide channelling [165], translocation of metabolites involved in oxidative phosphorylation [166,167] and mitochondrial transport [168,169]. Tubulin tethering to mitochondria has also been shown to play structural and functional roles in striated muscle homeostasis and disease [159,170]. Myofibrils can also more directly regulate myocyte metabolism through their interaction with mitochondria. The mitochondria failed to localize around myofibrils, where high energy is needed in mice lacking muscle LIM protein (MLP), suggesting that cytoskeletal MLP may be part of an energy-sensing mechanism [171]. Furthermore, a recent study reported that mutating key myofibrillar genes, such as *Actn2* and *Myh6*, disrupted mitochondrial maturation, thereby suggesting that sarcomere organization is upstream of mitochondrial maturation [95].

Mitochondria are positioned between lined myofibrils and mechanically elongated by the myofibrils. The geometry of mitochondria also influences the interaction between sarcomeres and thick filaments within the muscle cell [172]. Myofibril and mitochondria morphogenesis are intimately linked in *Drosophila* muscles. Mitochondrial over-fusion during myofibril assembly prevents mitochondrial intercalation in flight muscles, thereby causing the shift of muscle-specific sarcomeric proteins [173]. Moreover, conditional knockout of *Drp1*, a mitochondrial fission factor, in cardiomyocytes led to cardiac dysfunction [174]. Additionally, muscle-specific Drp1 loss induces muscle wasting and weakness owing to intracellular signalling changes induced by mitochondrial morphological changes [175]. Contrarily, mutations that affect the mitochondria, such as *Mfn1/2* and *Tfam*, had minor effects on sarcomere organization but altered cardiomyocyte proliferation [95,176].

Collectively, mitochondria are important organelles that maintain sarcomere function, but they are also functionally regulated by sarcomeres, and both work side by side to maintain the cardiomyocyte functions. However, the molecular dialogue in the context of maturation between the sarcomere and mitochondria remains to be elucidated.

### (b) Transverse tubules

The well-developed t-tubules are one of the key differences between neonatal and adult cardiomyocytes [177]. The t-tubules are complex and interconnected invaginations of the sarcolemmal membrane, which propagates action potential through the cardiomyocyte and initiates excitation-contraction coupling [178]. The three-dimensional model results revealed that the t-tubules in the heart project not only transversely but also in different directions, and their diameters range from 20 to 450 nm [179]. T-tubules of the skeletal muscle are much smaller, with diameters ranging from 20 to 40 nm [180,181]. Bridging integrator 1 (BIN1) is one of the t-tubule formation regulators, and BIN1 reduction suppressed the t-tubule invaginations, but overall cardiomyocyte morphology remained intact [182]. Caveolin-3 (Cav3) is another regulator that interacts with BIN1 [183]. The deletion of *Cav3* resulted in cardiomyocyte hypertrophy, although t-tubule formation was not completely abolished [184]. Emerging evidence suggests a possible role of sarcomeres in t-tubule formation and maturation. T-tubules are anchored to sarcomeres, and cardiomyocytes exhibited defective t-tubule organization with depleted α-actinin [96]. Moreover, an actin-binding protein, nexilin, that stabilizes the sarcomere was recently shown as important for t-tubule formation [185,186]. Similar to mitochondria, morphological changes owing to the lack of Drp1 cause structural abnormalities in the t-tubule [175]. These results suggest a close relationship between mitochondrial morphology and t-tubules.

### (c) Sarcoplasmic reticulum

The sarcoplasmic reticulum (SR) is an organelle found within the muscle cells and is similar to the smooth endoplasmic reticulum in other cells. SR constitutes major intracellular calcium storage in striated muscles and plays an important role in regulating excitation-contraction coupling and intracellular calcium concentrations during contraction and relaxation. SR has two forms, namely, longitudinal SR (l-SR) and junctional SR (j-SR) [187]. The repetitive close apposition between j-SR and t-tubules is essential for efficient excitation-contraction coupling [188]. The latest study implicated the junctophilin-2 on SR tethers t-tubules [189]. Upon action potential, Ca^2+^ microdomains, which are generated in synchrony at the interface between j-SR and t-tubules, underlie an immediate increase in cytosolic Ca^2+^ concentration, ultimately responsible for cell contraction during systole [190–192]. This process requires mitochondrial involvement, the main energy source of cardiomyocytes. Interestingly, mitochondrial distribution is highly ordered and strategically juxtaposed with SR in adult cardiomyocytes [188,193–195]. Mitochondria take up Ca^2+^ and modulate ATP synthesis according to the specific cardiac workload when Ca^2+^ release sites are close together [196–198]. Increased mitochondrial Ca^2+^ is pivotal in mitochondrial dehydrogenase activation and thus is essential for adjusting ATP production to cardiac needs during contraction [199,200]. Mitochondrial Ca^2+^ oscillates synchronously with cytosolic Ca^2+^ in cardiac cells, and mitochondrial Ca^2+^ handling rapidly adapts to inotropic or chronotropic inputs [201]. Thus, Ca^2+^ and ATP, which are required for sarcomere contraction, are regulated by t-tubules, SR and mitochondria interactions. SR mediates the growth of mitochondria and myofibrils at the intercalated disc in an electron microscopy study [202]. Furthermore, another study revealed that the close association of SR, mitochondria, and t-tubules was disrupted in heart failure [192]. While SR and mitochondria are in direct contact with sarcomeres, functions between them are unclear; hence, more studies are required for further understanding of the interplays across organelles and sarcomeres.

### (d) Nucleus

The nucleus also interacts with sarcomeres. The nuclear membrane is vital in maintaining genome stability and overall cellular dynamics. The linker of the nucleoskeleton and cytoskeleton (LINC) complex, which is composed of nesprins and Sad1p-UNC-84 domain 1 and 2 (SUN1/2) proteins, anchors actin filaments, intermediate filaments and microtubules to the nucleus. SUN proteins are also linked to the nuclear lamina, a structure near the inner nuclear membrane. The nuclear lamina directly interacts with chromatin to form lamina-associated chromatin domains [203]. The nuclear architecture is maintained through a balance between intermediate filaments and microtubules in the cytoplasm of cardiomyocytes, and the nuclear lamina counteracts the forces from the cytoplasm [204]. A key nuclear lamina protein is nuclear Lamin type A, which is encoded by the *LMNA* gene, and *LMNA* mutations cause DCM, conduction defects and ventricular arrhythmias [205]. LMNA protein disruption disorganizes the nuclear lamina to increase mechanical stress susceptibility. The conformational changes of the nuclear lamina not only result in impaired signalling from extracellular and cytoplasmic domains but also disrupt chromatin structure, directly affecting the gene transcription. Furthermore, similar to proto-costamere, the LINC complex recruits ZASP to the cytoplasmic side of the nucleus before sarcomere assembly and is also vital for sarcomere assembly and stability [206,207].

Sarcomere-related proteins also serve as direct signalling molecules to the nucleus from sarcomeres [208]. Some transcriptional factors and chromatin modifiers, e.g. nuclear factor of activated T cells 3, core-binding factor *β*, muscle-specific RING finger proteins and SMYD1, localize to sarcomeres and translocate to impact gene transcription upon defined stimuli [209]. Interestingly, sarcomere proteins (e.g. MLP, tropomyosin and troponin proteins) contain nuclear localization signals and may affect the nuclear structure or transcription [208], which remains to be further elucidated.

## 7. Summary and future perspectives

The sarcomere is the contractile unit of cardiomyocytes. Optimal structure and function are required for it to efficiently beat throughout our life. Therefore, it goes through a complex assembly and maturation process to reach its adult-like structure and function. The sarcomere and other organelle maturation processes in cardiomyocytes should progress hand-in-hand but is not fully understood. A comprehensive approach is needed to study and recapitulate the regulatory network of molecular interactions that occur during development *in vivo*. Herein, we focused on sarcomere maturation for its functional importance, regulatory machinery and central role in regulating other cardiomyocyte organelle maturation.

Many molecules have been reported to regulate the process of sarcomere development and maturation. Several transcription factors, such as SRF, GATA4, GATA6 and their modulators, work to regulate the levels of sarcomere gene expression on the gene expression levels. Nuclear receptors, such as THRs, ERRs, PPARs and glucocorticoid receptors, exert similar functions when activated. In the next step, RNA-binding proteins modulate alternative splicing, and miRNAs modulate the translation from transcripts. Outside the nucleus, sarcomere assembly machinery takes place for sarcomere formation and maturation. Sarcomere assembly and maturation is a dynamic, orchestrated process that reflects the cellular state and signalling environment.

Some molecules may have interchangeable functions given the complex regulatory network required for sarcomere maturation. Hence, dysfunction or deficiency of any of the aforementioned molecules can result in a wide range of manifestations. This was shown via animal models in which inflicting cardiac-specific mutations in sarcomere-regulating genes caused phenotypes ranging from apparently normal to heart failure and premature death. Consequences of failed sarcomere maturation and mutations in sarcomere protein genes range from asymptomatic to full-blown cardiomyopathy, heart failure and death in humans.

Having said that, many sarcomere maturation aspects are not well-understood. More research is needed to further illuminate the mechanisms by which the sarcomere acquires its mature phenotype. To this end, pluripotent stem cell-derived cardiomyocytes (PSC-CMs), either from embryonic or induced PSCs, are appealing tools to study the sarcomere maturation process and failed maturation consequences. Recent studies that used PSC-CMs noticeably support the importance of environmental cues for cardiomyocyte maturation, including extracellular matrix [73], appropriate culture substrate stiffness [210], nuclear receptors [139,211] and nutrients [212]. With the progress, PSC-CMs can finally be used as a model for studying the sarcomere maturation process because PSC-CMs are better at tracing the entire process compared to the snapshots from the heart. More importantly, human PSC-CMs eliminate the hurdle of species-related physiological differences encountered when animal models are used.

Emerging pieces of evidence are pointing towards the sarcomere as being not only the force generator but also the master regulator of cardiomyocyte maturation in contrast with the previous presumption that mitochondria regulate cardiomyocyte maturation. Such a hypothesis is evident in the underdevelopment of organelles, such as the mitochondria and t-tubules, when myofibrillar genes are mutated. This new understanding should gear our efforts towards studying sarcomeres as a signalling hub that regulates cardiomyocyte maturation and function. Significant progress was seen on how sarcomeres and cardiomyocytes mature; however, a lot remained to be elucidated. For example, cardiomyocytes have unique rectangular morphology; however, it remained unknown how they become rectangular. Z-discs are anchored by costamere (figures 1*d* and 3); thus, the extension of the cell membrane by the addition of sarcomeres to myofibrils is a possibility. Recent advances in new technology, such as live imaging, may provide further insights. The interplays between sarcomeres and other organelles have pivotal roles in cardiac diseases and studying how sarcomeres acquire their mature forms and the maintenance machinery can be translated to the understanding of how they work in adult hearts to maintain the cardiomyocyte physiology and play in diseased conditions.

## Data Availability

This article has no additional data.
